# Cytotoxic, analgesic and anti-inflammatory activity of colchicine and its C-10 sulfur containing derivatives

**DOI:** 10.1038/s41598-021-88260-1

**Published:** 2021-04-27

**Authors:** Joanna Kurek, Krzysztof Myszkowski, Irena Okulicz-Kozaryn, Agnieszka Kurant, Ewa Kamińska, Michał Szulc, Błażej Rubiś, Mariusz Kaczmarek, Przemysław Ł. Mikołajczak, Marek Murias

**Affiliations:** 1 Department of Bioactive Products, Faculty of Chemistry, Adam Mickiewicz University, Uniwersytetu Poznańskiego 8, 61-614 Poznan, Poland; 2 Department of Toxicology, Poznań University of Medical Sciences, Dojazd 30, 60-631 Poznan, Poland; 3 Department of Pharmacology, Poznań University of Medical Sciences, Rokietnicka 5a, 60-806 Poznan, Poland; 4 Department of Clinical Chemistry and Molecular Diagnostics, Poznań University of Medical Sciences, Przybyszewskiego 49, 60-355 Poznan, Poland; 5 Department of Cancer Immunology, Chair of Medical Biotechnology, Poznan University of Medical Sciences, Garbary 15 Str, 61-866 Poznan, Poland; 6 Gene Therapy Laboratory, Department of Cancer Diagnostics and Immunology, Greater Poland Cancer Centre, Garbary 15 Str, 61-866, Poznan, Poland

**Keywords:** Cancer, Chemical biology, Drug discovery, Molecular biology

## Abstract

10-Alkylthiocolchicines have been obtained and characterized by spectroscopic methods and their biological activities as: cytotoxic, anti-inflammatory and analgesic activities have been tested. Cytotoxic activity against SKOV-3 ovarian cell line for 10-alkylthiocolchicine analogues was reported and tested compounds showed to be more active than commonly used doxorubicin. Some of tested C-10 alkylthiolated colchicines have been found to exhibit cytotoxicity at levels comparable to that of the natural product—colchicine. 10-Methylthiocolchicine has IC_50_ = 8 nM and 10-ethylthiocolchicine has IC_50_ = 47 nM in comparison to colchicine IC_50_ = 37 nM. Moreover for 10-alkylthioderivatives apoptosis test, cyclin B1 and cell cycle tests were performed. 10-*n*-Butylthiocolchicine was tested for anti-inflammatory and analgesic activities it showed to produce analgesic rather than anti-inflammatory effect.

## Introduction

**Figure 1:**
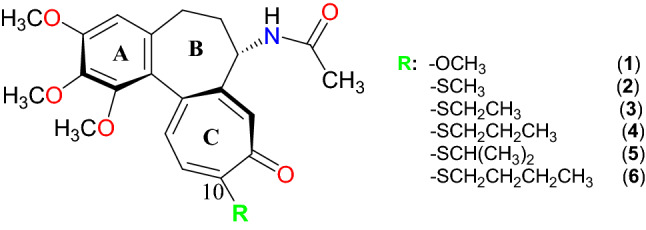
Colchicine **1** and its derivatives **2–6**.

Colchicine **1** (Fig. 1) is a naturally occurring alkaloid in the *Liliaceae* family^1,2^. It is a well-known bio-active compound, possessing antimitotic and anti-inflammatory activity. Colchicine inhibits of microtubule polymerisation and this antimitotic action was fully explained in 2004^3^. Colchicine due to its anti-inflammatory activity is useful as a drug during gout attacks. Its activity is exerted via inhibition of mainly proinflammatory cytokines and inflammasome (key players in inflammatory signalling pathways). Secondly, colchicine also inhibits neutrophil migration^4^. Colchicine is usually useful drug also for familiar Mediterranean fever (FMF), Bechet’s disease, chondrocalcinosis and other microcrystalline arthritis, but as cytotoxic agent has limited application because of its high toxicity.

Many attempts have been made to synthesize colchicine derivatives for possible medical purposes, which could be less toxic and more potent than colchicine. 10-Alkylthiocochicines have been obtained in different synthetic conditions^4–8^. One of the well-known derivatives is 10-methylthiocolchicine **2** because it has a good therapeutic index and is less toxic than **1**^4,9–11^. On this basis usually 10-methylthiocolchicine has to be modified to obtain more active compounds as cytotoxic agents, which possess superior pharmacological properties, accompanied by decreased toxicity, which makes these derivatives important compounds for anti-inflammatory and anticancer therapy^12,13^. Compounds **2–6** have been modified for searching new active derivatives^14^. Cytotoxic activity against SKOV-3 cancer cell line and also anti-inflammatory and analgesic activities of these compounds have not been tested yet.

The aim of this study was to find out if 10-alkylthiocolchicines showed some cytotoxic activity against SKOV-3 ovarian cancer cell line. For other tests (apoptosis test, cyclin B1 and cell cycle tests) were chosen colchicine **1** and derivatives with the shortest (CH_3_S–) **2** and the longest (*n*-C_4_H_9_S–) substituent **6** among tested compounds. Since colchicine possesses anti-inflammatory activity so **6** was tested for its anti-inflammatory and analgesic activity.

## Results

10-Alkylthiocolchicines were obtained by reaction of colchicine with respective sodium alkylthiolates^5^. In the present study analogues **2**–**6** (Fig. 1) were evaluated for cytotoxicity towards SKOV-3 ovarian human cancer cell line, effect on cell cycle and apoptosis. 10-*n*-Buthylthiocolchicine **6** was also tested as a possible anti-inflammatory and analgesic agent.

**Figure 2:**
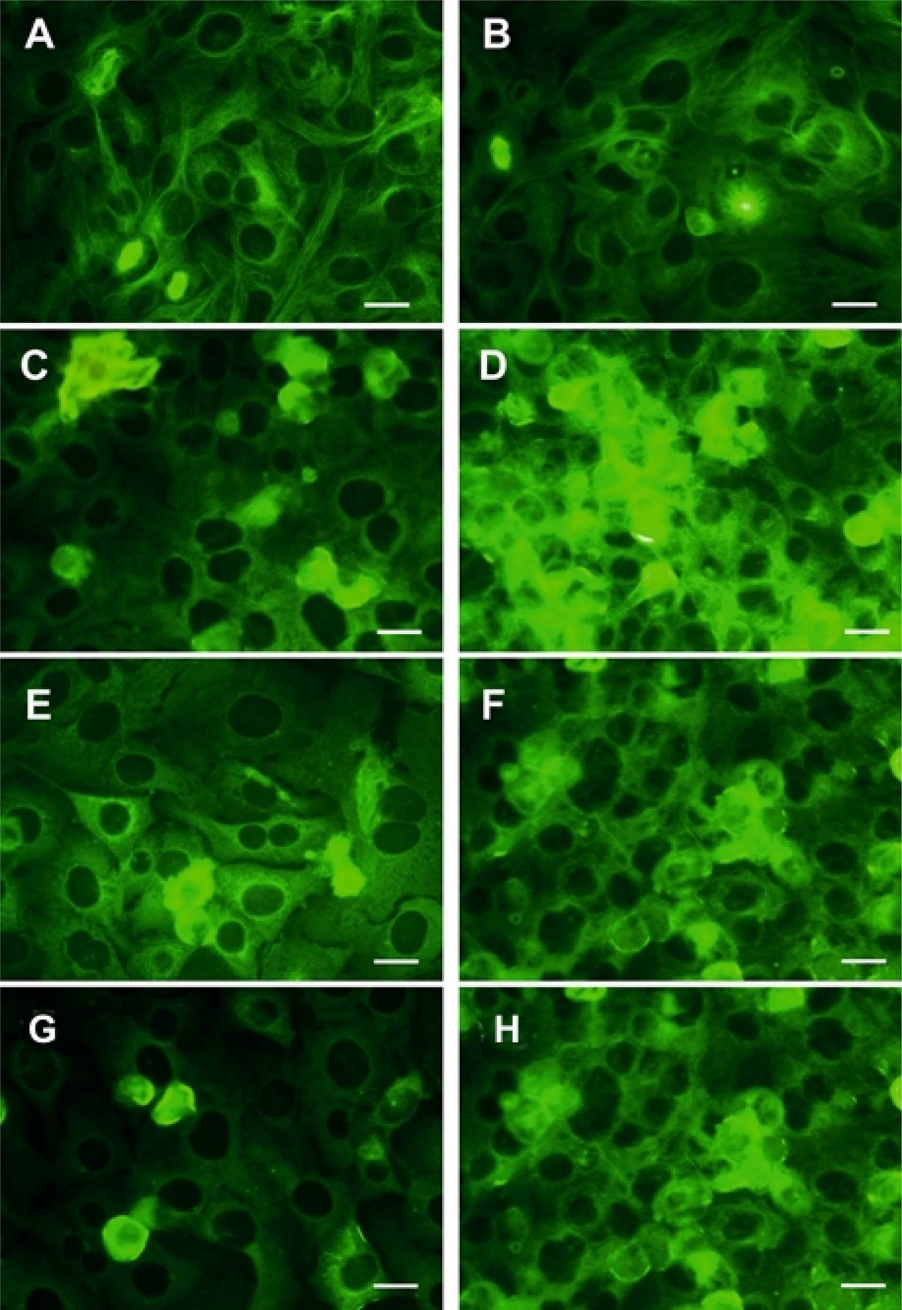
Effect on α-tubulin polymerization in SKOV-3 cells incubated with tested compounds for 24 h. (**A**, **B**) control, (**C**,**D**) **1** 0,1 μM and 1.0 μM respectively; (**E**,**F**) compound **2** 0,1 μM and 1.0 μM respectively; (**G**,**H**) compound **6** 0.1 μM and 1.0 μM (bar, 10 μm; FITC-conjugated primary antibody).

As it is presented in Fig. 2 in control cells a diffuse microtubule network is present, while in cells treated with colchicine and compounds **2** and **6** condensation and fragmentation of tubulin can be observed.

Cytotoxic activities of these semisynthetic alkaloids were previously screened against DLD1, LoVo, MCF-7 and MDA MB-231 cell lines^5^. Some derivatives of 10-alkylthiocolchicines were also tested against cancer cell lines and showed to be active^14^. The tested compounds showed potent cytotoxic activity against SKOV-3 ovarian cancer cell line with the calculated IC_50_ values significantly lower than IC_50_ measured for the commonly known anticancer drug doxorubicin (Table 1). Cytotoxicity of colchicine and the compounds **2** and **6** against proliferating and growth arrested cells SKOV-3 human cancer IC_25_ (24 h) and IC_50_ (72 h) are given in Table 2.

**Table 1:** The IC_50_ values of compounds tested against SKOV-3 ovarian cancer cell line after 72 h incubation; **1**—positive control.

Compound	IC_50_ [µM]
**1**	0.037 ± 0.004
**2**	0.008 ± 0.001
**3**	0.047 ± 0.005
**4**	0.362 ± 0.028
**5**	0.332 ± 0.030
**6**	0.780 ± 0.036
Doxorubicin	3.339 ± 0.163

**Table 2:** Cytotoxicity of colchicine and the compounds **2** and **6** against SKOV-3 human cancer cell line 24 h; **1**—positive control.

	IC_25_[µM]—24 h		IC_50_[µM]—72 h
	Proliferating	Growth arrested	Growth arrested
**1**	> 10	> 10	0.052 ± 0.008
**2**	0.010 ± 0.075	> 10	0.009 ± 0.002
**6**	1.110 ± 1.156	> 10	1.845 ± 0.128

Data for proliferating cells after 72 h are given in Table 1.

**Figure 3:**
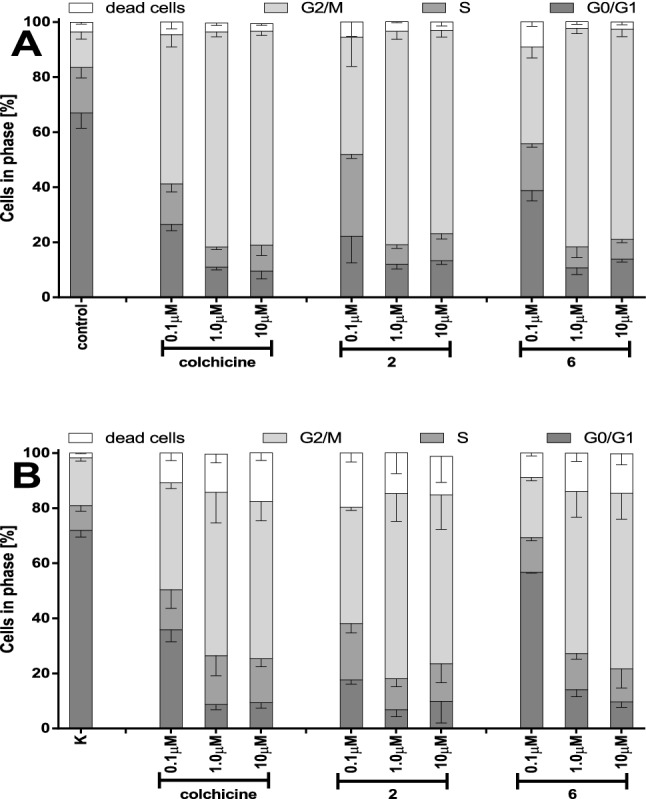
Effect of tested compounds on cell cycle distribution in SKOV-3 ovarian cancer cells. Cells were treated with tested compounds: colchicine **1**, **2** and **6** at concentrations 0.1 μM, 1.0 μM, and 1.0 μM for 24 h (**A**) and 72 h (**B**). After incubation cells were stained with propidium iodide and analyzed using flow cytometry. Statistical significances are marked with asterisks (*) p < 0.05; (**) p < 0.01; (***) p < 0.001.

It may be easily noted that the cytotoxic effect of tested compounds depends on the length of alkylthio chain. Therefore for further tests compounds **2** and **6** possessing the shortest and the longest alkylthio chains were selected. As it is presented in Fig. 3 strong block in G_2_M phase was observed in cells incubated with tested compounds: colchicine **1**, **2** and **6** for 24 h and was followed by increased percentage of dead cells in samples incubated for 72 h. Cell cycle analysis of SKOV-3 cell line cultured in the presence of colchicine **1**, 10-methylthiocolchicine **2** and 10-*n*-butylthiocolchicine **6** at concentrations of 0.1 µM, 1.0 µM and 10 µM after 24 and 72 h are included in supplementary file Fig. S3.

**Figure 4:**
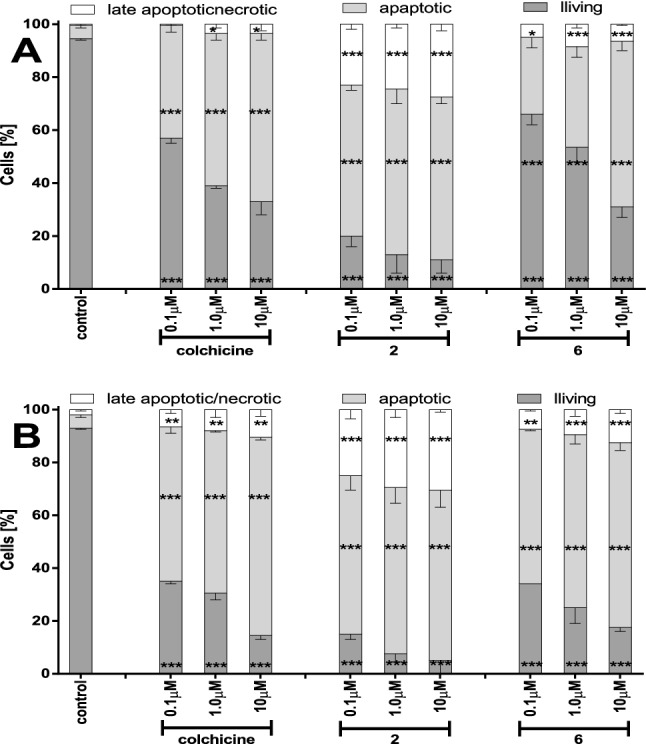
Apoptosis induction in SKOV-3 treated with compounds: **1**, **2** and **6** at concentrations 0.1 μM, 1.0 μM, and 1.0 μM for 24 h (**A**) and 72 h (**B**). After incubation cells were stained with stained Hoechst 33,258 and propidium iodide and evaluated using fluorescence microscopy. Statistical significances are marked with asterisks (*) p < 0.05; (**) p < 0.01; (***) p < 0.001.

In Fig. 4 is presented apoptosis induction in SKOV-3 cells treated with tested compounds: **1**, **2** and **6** in three different concentrations: 0.1 μM, 1.0 μM, and 1.0 μM and incubated with tested compounds for 24 h and 72 h.

**Figure 5:**
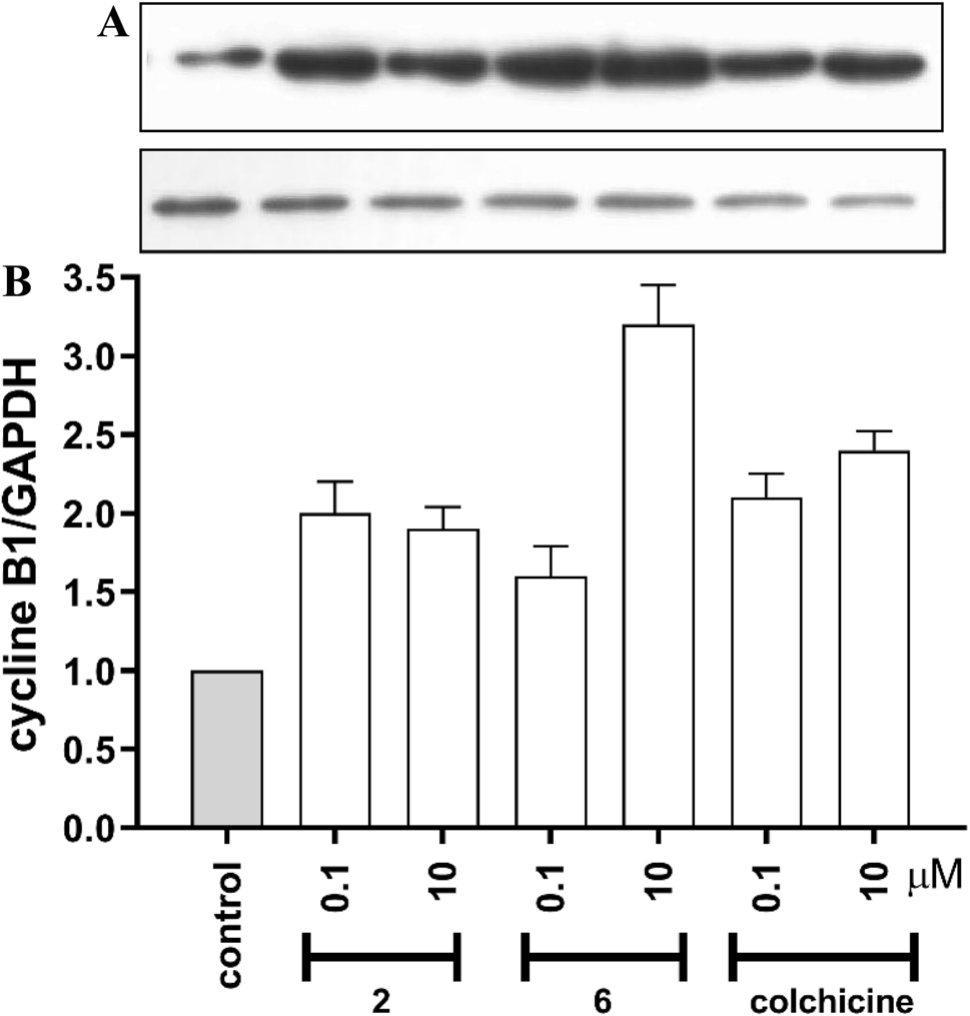
Impact of tested compounds on Cyclin B1 expression in SKOV-3 cells. Cells were treated with tested compounds: colchicine, **2** and **6** at concentrations 0.1 μM, 10.0 μM for 24 h. All tested groups were significantly different from control group, p < 0.001 (**A**) Western blot, (**B**) corresponding results of densitometric analysis.

These observations are consistent with microscopic observations of α-tubulin immunocytochemically stained in SKOV-3 cells. Western blot analysis showed that these changes were also accompanied with increased level of cyclin B1, which normally arises in M phase (Fig. 5 and Fig. S2). The highest level of cyclin B_1_ was measured in cells incubated with compound **1**, **2** and **6** what corresponds with changes observed in cell cycle distribution measure by flow cytometry.

**Figure 6:**
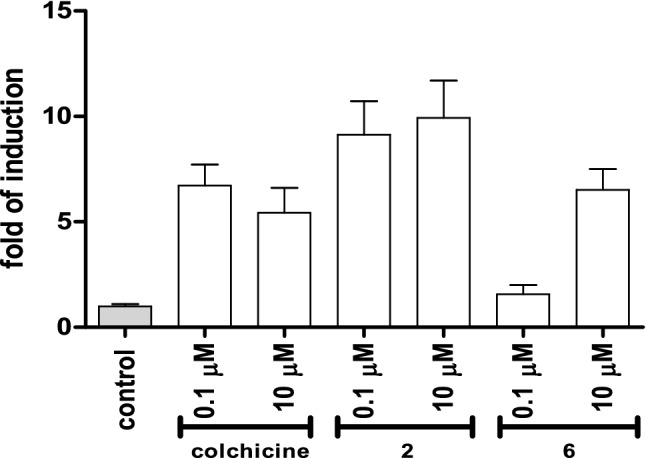
Impact of tested compounds: colchicine, **2** and **6** on Caspase 3/7 activation in SKOV-3 cells. Cells were treated with tested compounds at concentrations 0.1 μM, and 10.0 μM for 24 h. All tested groups were significantly different from control group, p < 0.001, except group treated with compound 6 at concentration 0.1 µM, p < 0.01.

The abnormal activation of cyclin B_1_ is usually connected with cell death type called mitotic catastrophe, which is usually, at early stage, caspase independent. In order to further explain mechanism of cell death induced in SKOV-3 ovarian cancer cells by tested compounds the activation of caspase 3/7 was assayed, (Fig. 6). Caspase-3 belongs to executioner caspases and is activated in both: intrinsic and extrinsic type of apoptosis.

In tested cells strong activation of caspase-3 was measured, except cells incubated with compound **6** used at concentration 0.1 µM. These results were consistent with results obtained in ELISA assay which allows relative quantification of histone-complexed DNA fragments (mono- and oligonucleosomes) out of the cytoplasm of cells after the induction of apoptosis or when released from necrotic cells.

**Figure 7:**
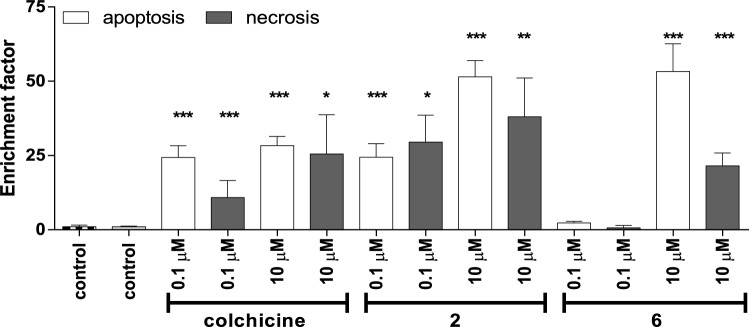
Effect of tested compounds: colchicine, **2** and **6** on apoptosis (white bars) and necrosis (grey bars) assayed using ELISA in SKOV-3 cells after 24 h of incubation. Statistical significances are marked with asterisks (*) p < 0.05; (**) p < 0.01; (***) p < 0.001.

This assay showed relatively high percentage of necrotic changes in cells incubated with compound **2** (Fig. 7). The morphological changes were therefore further evaluated in cells stained with Hoechst 33258 and propidium iodide. This assay allow to discriminate between viable, necrotic and apoptotic cells. More intensive staining with Hoechst 33258 was interpreted as a consequence of nuclear condensation while treatment-related impairment of the membrane function was detected by PI staining, which was concentration- and exposure-dependent. Treatment with **2** or **6** resulted in profoundly disturbed membrane permeability, indicating a higher contribution of necrosis induction.

**Figure 8:**
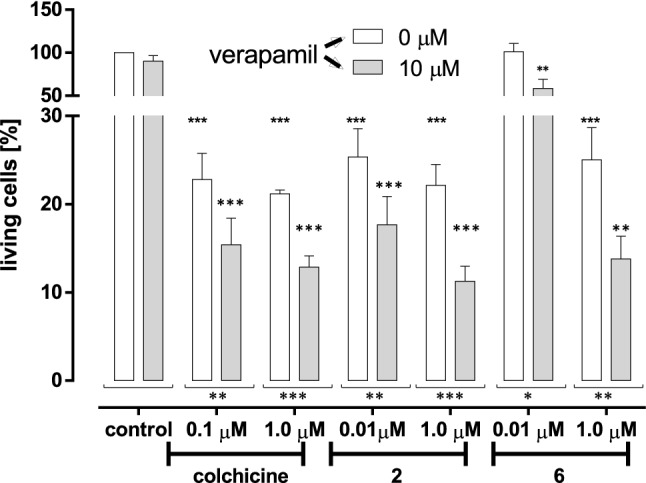
Impact of verapamil (ABCB_1_ inhibitor) on cytotoxic activity of tested compounds: colchicine, **2** and **6**. Statistical significances between groups treated without verapamil are marked with asterisks (*) p < 0.05; (**) p < 0.01; (***) p < 0.001. Statistical significances between groups treated with verapamil are marked with asterisks (*) p < 0.05; (**) p < 0.01; (***) p < 0.001. Statistical significances between groups treated with the same concentration of tested compound but with or without verapamil are marked with asterisks (*) p < 0.05; (**) p < 0.01; (***) p < 0.001. (ANOVA F(3, 33) = 16.5; p = 0.0000).

Finally, the impact of ABCB_1_ activity on cytotoxic potential of tested compounds was evaluated, (Fig. 8). ABCB_1_ (also known as multidrug resistance protein 1-MDR1) is an efflux transporter facilitating transport of anticancer drugs out of the cell and therefore rendering cancers cells multidrug resistant. In this assay verapamil was used as ABCB_1_ inhibitor.

As it is shown in Fig. 8 simultaneous incubation of SKOV-3 cells with tested compounds and verapamil increased their cytotoxic potential suggesting improve cytotoxic activity of tested analogues by simultaneous use of ABCB_1_ inhibitors.

### Anti-inflammatory and analgesic study

Anti-inflammatory and analgesic activities of 10-*n*-buthylthiocolchicine **6** were tested. The study was performed using 5 groups of rats (n = 6–8 in each group). Two doses of 1.0 and 10.0 mg/kg b.w. (i.p.) of the compound were given to the animals. The study was performed using 5 groups of rats (n = 6–8 in each group). Two doses of 1.0 and 10.0 mg/kg b.w. (i.p.) of the compounds were given to the animals. Morphine and indomethacine in this study were chosen as reference standard drugs possessing quite different mechanism of action: indomethacine—nonsteroidal anti-inflammatory agent, nonselective inhibitor of COX-1 and COX-2, morphine—opioid agonist acting mainly via μ (MOR) receptor in brain, spinal cord and ends of peripheral of C fibres. The dose and route of administration e.g. morphine (5.0 mg/kg b.w., s.c.) and indomethacine (10.0 mg/kg b.w., i.p.) were used according to previous studies^15–17^. The carrageenan-induced acute inflammation in rats is characterized by hyperalgesia, edema, and strong redness. Maximum of described effects typically is observed at 3–6 h after the injection^18,19^. In this experiment carrageenan was administered in the dose of 2000 μg (0.2 mL of 1% solution)^20^.

**Figure 9:**
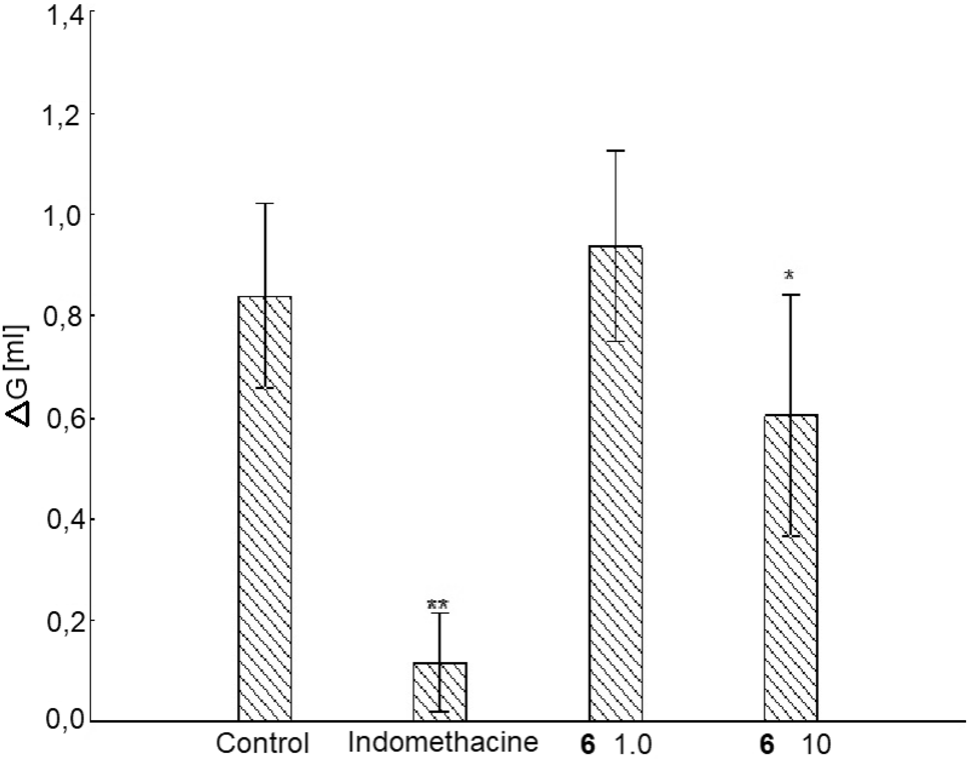
Influence of **6** and indomethacine on anti-inflammatory activity in rats. Values are mean ± SEM, ΔG—value expressing change in paw’s thickness against baseline (before inflammation) after 3 h, **6 (**6–1.0) a dose of 1.0 mg/kg, **6 (**6–10) a dose of 10.0 mg/kg, **, *—significant difference vs Control group; p < 0.05 or p < 0.1, respectively.

In studies on the influence of the tested compounds on anti-inflammatory activity in the experimental system, a statistically significant general variability was demonstrated (one way ANOVA F(3,33) = 16.5; p < 0.0001). Further analysis (Duncan post hoc test) allowed to determine the anti-inflammatory effect in relation to the control group, Fig. 9. This was characterized by administration of the analyzed compound only at a dose of 10.0 mg/kg, but the results did not show a strong significance when compared with the corresponding control values (p < 0.1). In this condition the reference drug-indomethacine produced more pronounced and significant effect (p < 0.05).

**Figure 10:**
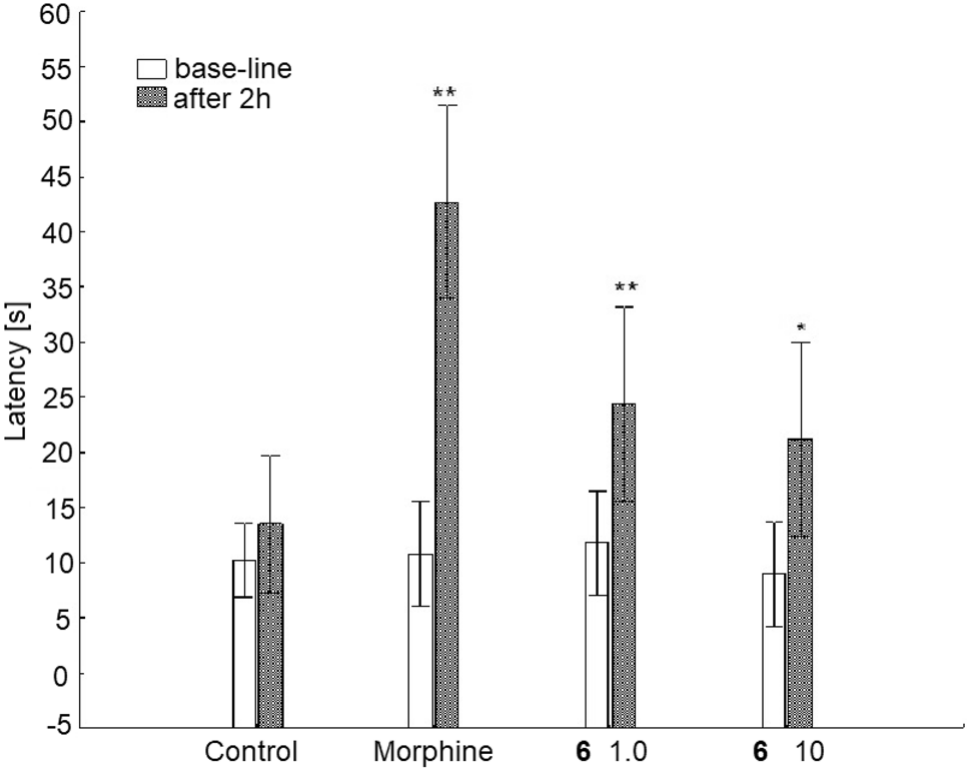
Influence of 6 and morphine on analgesic activity using tail-flick test. Values are mean ± SEM, **6 (**6–1.0) a dose of 1.0 mg/kg, **6 (**6–10) a dose of 10.0 mg/kg, **, * significant difference vs Control group; p < 0.05 or p < 0.1, respectively.

On the contrary, analyzing the analgesic activity of **6** the stronger effects were observed, Fig. 10. Analysis of the interaction of both factors (existence of variation between the means and the effect of time) studied in this test indicated the importance of the action of both effects (ANOVA, interaction F(3,36) = 13.4; p = 0.0000). It was observed that the morphine showed a significant effect when compared with the control group (p < 0.05), whereas the compound **6** produced statistically significant effect only in lower dose when compared with the control group (p < 0.05).

## Discussion

Cytotoxic, analgesic and anti-inflammatory activities of 10-alkylthiocolchines have been tested. All tested compounds showed better cytotoxic activity against SKOV-3 cell line than commonly known cytotoxic agent doxorubicin. Cytotoxic effect of tested compounds depends on the length of alkylthio chain. These changes in blocked cell cycle were also accompanied with increased level of cyclin B1, which normally arises in M phase. Cytotoxic activity of tested analogues can be potentially improve by simultaneous use of ABCB_1_ inhibitor (verapamil) with incubation of SKOV-3 cells with tested compounds increased their cytotoxic.

It is known that gouty arthritis is characterized by increased hyperuricemia level and leads to the deposition of monosodium urate crystals within synovial joints and tissues, which produces an acute inflammatory attack with intense pain, swelling and skin reddening^21,22^. Colchicine and non-steroidal anti-inflammatory drugs (NSAIDs) (e.g., indomethacine), which are first-line treatments that inhibit this process clinically, are frequently used against acute gouty arthritis^23,24^. Nonetheless, the inevitable side effects involving gastrointestinal bleeding, gastrointestinal toxicity and renal toxicity of such pharmacological reagents restrict their further utilization^25,26^. Therefore searching new safer colchicine analogues seems to be interesting. It is known that in mice an LD_50_ (i.p.) for colchicine was established for a dose of 1.6 mg/kg^10^. It was to be expected that the compound **6** may have a similar toxicity. It turned out, however, based on our research it is known that for doses up to 20 mg/kg/day (i.p.) mice did not show any mortality or toxic effects during next 14 days (data not shown). This demonstrated that **6** has at least approx. 10 times less toxicity in comparison to **1** (colchicine). Further examination of acute toxicity at higher doses was impossible due to the limitations of its solubility.

10-*n*-Butylthiocolchicine **6** showed the analgesic activity rather than anti-inflammatory activity. The observed analgesic effect in tail flick test is interesting, although on the basis of our results it is not possible to explain the mechanism responsible for such action. It is known that tail-flick test represents a model of acute thermal pain and is a standard method for investigating nociception and analgesia, especially selective for centrally acting analgesics, with the measurement of the response to a brief, noxious stimulus which appears to be a spinal reflex, modulated by supraspinal inhibitory mechanism^20^. However, in this model also NSAIDs, which inhibit cyclooxygenase in peripheral tissues, thereby interfering with the mechanism of transduction in primary afferent nociceptors, can also produce analgesic effects^27^. There is a report that intracerebroventricular (i.c.v.) colchicine administration before morphine injection (i.c.v.) prevented the analgesic effect of morphine using tail-flick test^28^. Since, many investigators use colchicine to block microtubular transport of neurotransmitters including endogenous opioids^29^, therefore the hypothesis can be made that via different than opioid system the observed analgesic effect shown by **6** is produced. However, to confirm this possibility, further detailed studies are needed.

No a strong anti-inflammatory activity was obtained in comparison to the classical drug indomethacine what might be related to the need to use higher dosages of **6**. Unfortunately, it was not possible to inject **6** in higher doses due to limitation of its solubility in water. Although many authors demonstrated the anti-inflammatory activity of colchicine^30,31^, it was found that based on the dosages required to achieve a 50% suppression of carrageenan-induced acute inflammation (IC_50_), colchicine showed only 60% activity produced by indomethacine^32^, what is in line with the anti-inflammatory results produced by **6**. It should be noted, however, that it cannot be ruled out that the stronger anti-inflammatory effect of the test compound could be observed after prolonged use, as mentioned for by some authors for the action of colchicines^21^, thus further investigations pharmacological after chronic administration **6** would help in resolving this hypothesis**.**

Concluding, the obtained results regarding the anticancer, analgesic and week anti-inflammatory activities are interesting however, the mechanisms underlying the observed effects are unknown; hence further detailed studies are needed.

## Methods

General procedure for the synthesis of 10-alkylthiocolchicine analogues 2–6 was described in^5^. Purity of obtained compounds was checked by HPLC and compounds were pure > 95%.

### Measurements

The NMR spectra of 10-alkylthioderivatives **2–6** (0.07 mol L^−1^) were recorded in CD_3_Cl solutions using a Varian Gemini 300 MHz spectrometer. All spectra were locked to deuterium resonance of CDCl_3_. The ^1^H NMR measurements in CDCl_3_ were carried out at the operating frequency 300.075 MHz; flip angle, pw = 45^0^; spectral width, sw = 4500 Hz; acquisition time, at = 2.0 s; relaxation delay, d_1_ = 1.0 s; T = 293.0 K and using TMS as the internal standard. No window function or zero filling was used. Digital resolution was 0.2 Hz per point. The error of chemical shift value was 0.01 ppm. ^13^C NMR spectra were recorded at the operating frequency 75.454 MHz; pw = 60^0^; sw = 19,000 Hz; at = 1.8 s; d_1_ = 1.0 s; T = 293.0 K and TMS as the internal standard. Line broadening parameters were 0.5 or 1 Hz. The error of chemical shift value was 0.01 ppm. The ^1^H and ^13^C NMR signals were assigned for each species using one or two-dimensional (COSY, HETCOR, HMBC) spectra. Mass Spectrometry the EI (Electron Impact) mass spectra were recorded on a Waters/Micromass (Manchester, UK) ZQ mass spectrometer equipped with a Harvard Apparatus syringe pump. Elementary Analysis The elementary nalysis of all 10-alkylthiocolchicine derivatives was carried out on Vario ELIII (Elementar, Germany).

### Cytotoxic study

#### Cell culture

All chemicals used in cell culture experiments, were obtained from Sigma-Aldrich (St. Louis, Mo, USA) unless otherwise stated. Human ovarian adenocarcinoma SKOV-3 cells were obtained from the European Collection of Cells Cultures; ECACC, Salisbury, UK. The cells were cultivated in DMEM, supplemented with 10% FBS, 1% penicillin/streptomycin and 1% l-glutamine at 37 °C (Gibco Invitrogen Corp. Grand Island, NY, USA), in a humidified atmosphere containing 5% CO2.

#### Cytotoxicity assay

To investigate the effects of tested compounds on cell viability, cells were detached using trypsin, and seeded in 96-well plates at a density 2 × 104 cells/well. They were allowed to attach overnight and colchicine analogues added from the stock solution prepared in DMSO (100 mM/mL). The final concentration of DMSO in cell treatment solutions was less than 0.1%. Control cells were cultured under the same conditions with 0.1% DMSO. Cell viability was evaluated by MTT assay. Briefly, medium was removed from the wells and 170 µl of reaction solution containing 3-(4,5-dimethyl-2-thiazolyl)-2,5-diphenyl-2H-tetrazolium bromide solution (5 mg/mL PBS) in culture medium was transferred to each well. The cells were incubated for 2 h under cell culture condition. After incubation the plates were centrifuged 3 min and formazan was dissolved in 200 µL DMSO. The absorbance was measured at 570 nm using a plate reader (Biotek Instruments, Elx-800)^33^. The results are presented as the mean ± SD from two independent experiments. The concentration of colchicine analogues, which caused 50% cell growth inhibition (IC50) was determined was calculated using GraphPad Prism 6.0 (GraphPad Prism 5.00, GraphPad Software, San Diego California USA). Additionally MTT was used for tests employing Verapamil as multidrug resistance protein 1 (MDR-1) inhibitor^34^. The cells were incubated with and without tested compounds and verapamil at concentration 10 µM for 72 h after incubation cell viability was assessed as it is described above. Information for Figs. 3, 4, 5, 6, 7 and 8. Statistical significance between groups was assessed by Dunnett’s Multiple Comparison Test^33,34^.

#### Apoptosis mono- and oligonucleosomes measured using ELISA

Apoptosis was evaluated using commercially available kit (Cell Death Detection ELISAPLUS, Roche) according to manufacturer’s protocol. The assay allows to measure mono- and oligonucleosomes formed during apoptosis and is based on a quantitative sandwich-enzyme-immunoassay-principle. The SKOV-3 cells (2 × 10^4^) were incubated with tested colchicine analogues and the ELISA assay was performed after 24 h. Cells were lysed and supernatant was collected after centrifugation at 3000×*g* for 10 min, than the samples were placed in a streptavidin-coated microtiter plate and incubated with a mixture of anti-histone-biotin, anti-DNA-peroxidase and incubation buffer. After 2 h of incubation, unbound antibodies were removed by using washing buffer. For quantification of the nucleosomes the absorbance was measure at 405 nm (reference wavelength 492 nm) for this purpose Biotek Instruments, Elx-800 was employed. The results are presented as enrichment factor (EF) are shown as the means ± SD from two independent experiments^35^.

#### Apoptosis caspase 3/7 activity

SKOV-3 cells were seeded in a 96-well plate at a density of 2 × 10^4^ cells per well, and after 24 h they were treated with a vehicle (0.1% DMSO) of tested compounds at 0.125 μM and 0.250 μM concentrations. After 24 h the medium was removed and activity of caspase 3/7 was measured using a luminescent Caspase-Glo-3/7 assay kit (Promega, USA). The kit was used according to the manufacturer’s protocol. Luminescence was measured using plate reader Tecan Infinite 200 (Mannedorf, Switzerland)^36^.

#### Apoptosis—propidium iodide and Hoechst 33258 staining

The morphological changes in cells undergoing apoptosis and necrosis were investigated using the double staining with Hoechst 33,258 and propidium iodide. SKOV-3 cells were seeded at density 2.5 × 10^5^ cells per well in 12-well plates and incubated under cell culture conditions. Cells were exposed to tested compounds for 24 h, next cells were incubated for 24 h and 72 h and stained with mixture: Hoechst 33258 (10 µg/mL)/propidium iodide (10 µg/mL) for 30 min at temperature 37 °C. Then, cells were washed twice, with PBS, fixed with 4% paraformaldehyde solution and incubated for 30 min at room temperature. Plates were again washed twice with PBS and 200 µL PBS was added to each well. Cells were evaluated under inverted microscope (Nikon Eclipse TS100). Cells were differentiated into one from three categories: viable, apoptotic and necrotic/late apoptotic population. The results are presented as mean values ± SD from two experiments^37^.

#### Immunostaining

The changes in cytoskeleton after colchicine analogues treatment were evaluated by fluorescence microscope using α-tubulin-FITC antibody. The SKOV-3 cells (seeded at density 2.5 × 10^5^) were fixed with 4% formaldehyde in phosphate buffer saline for 15 min at room temperature. Then, cells were washed twice; with PBS and 1% Triton X-100 was used as permeabilizing agent. Subsequently, cells were incubated with a blocking solution (1% BSA in PBS) for 1 h at room temperature. After washing, twice with PBS, cells were stained using anti-α-tubulin-FITC (1:50) antibody overnight at 4 °C. Images were captured using a fluorescence microscope (Nikon Eclipse TS100 microscope with attached fluorescence unit model C-SHG and digital camera DS-SMc)^38^.

#### Cyclin B1

SKOV-3 cells were seeded in 10 cm Petri dishes and cultured overnight. Then, cells were incubated with tested compounds at concentrations: 0.25 μM; 0.5 μM for 24 h, 48 h and 72 h. Cells were lyesed using RIPA buffer containing proteases inhibitors for 30 min at 4 °C. Lysates were centrifuged and supernatants were assayed for protein content using Bio-Rad DC Protein Assay Kit (Hercules, CA, USA) and 100 μg of each extract were loaded onto SDS–PAGE gels. Western blotting was performed by standard procedure using a PVDF membrane (Pierce Biotechnology, Rockford, USA). The following antibodies were used anti-cyclin B from Santa Cruz Biotechnology. The concentration in the blotting solution of each primary antibody was 1 μg/ml. After washing membranes were exposed to secondary antibody conjugated with HRP for 1 h. The proteins were visualized using SuperSignal West Pico Chemiluminescent Substrate and CL-X Posur film (Pierce Biotechnology, Rockford, USA). The optical density (Arbitrary Units) of the bands was measured using LabWorks software (UVP, Upland, CA)^39^.

#### Cell cycle

To determine the distribution of cells in different phases of the cell cycle the propidium iodide staining and flow cytometry were employed^40^. The SKOV-3 cells were seeded in 6-well plate at density 5 × 10^5^ cells per well and left to attach overnight. The cells were exposed to tested compound at concentrations: 0.25 μM; 0.5 μM; 1 μM and incubated for different time points: 24 h, 48 h and 72 h. Cells were collected by trypsinization, washed twice with PBS and fixed by 70% ethanol (Avantor Performance Materials S.A. Gliwice, Poland) at 4 °C. After 30 min, the cells were spun down (3000 rpm) and cell pellets were rinsed twice with PBS. The cells were then re-suspended in PBS containing 50 μg/ml of propidium iodide in the presence of 100 μg/ml RNase A and incubated for 30 min, at room temperature and protected from light. Cell cycle analysis was determined by using FACS Calibur flow cytometer (Becton & Dickinson, USA.)^40^.

### Analgesic study

#### Animals

Experiments were performed on male Swiss mice (21–39 g) and male Wistar rats (180–220 g) at the initiation of the experimental procedure. The healthy, pathogen free mice and rats were obtained from Laboratory Animals Supplier (Ogrodowa 18, 05–840 Brwinów, Poland). Next, the animals were acclimatized for at least 1 week prior to use. The total number of rats at the beginning of the experiment was n = 32, whereas number of mice was n = 30. The animals housed in controlled room temperature (20 ± 0.2 °C) and humidity (65–75%) under a 12 h: 12 h light–dark cycle (lights on 7 a.m.), kept in groups of 5 mice and 2–3 rats in light plastic cages (40 cm × 30 cm × 15 cm, for mice and 60 cm × 40 cm × 20 cm, for rats, respectively) and had a free access to standard laboratory diet (pellets-Labofeed B) and tap water in their cages^41^. The experiments with animals were performed in accordance with Polish governmental regulations (Animal Protection Act, Poland-Dz.U. (Journal of Laws)—Dz.U.05.33.289,2005) and in compliance with the ARRIVE guidelines (http://www.nc3rs.org.uk/page.asp?id=1357). The study was conducted in accordance to ethical guidelines for investigations of experimental pain in conscious animals and the study protocol was approved by the Local Ethics Committee of the Use of Laboratory Animals in Poznań, Poland.

#### Substance: 10-*n*-butylthiocolchicine

The compound **6** was given intraperitoneally (i.p.) in doses of 1.0; 2.0; 10.0 and 20.0 mg/kg (acute toxicity study) to mice or 1.0 and 10.0 mg/kg (pharmacological part of the study) to rats dissolved in water for injection in a volume of 1.0 or 10.0 mg/mL (respectively). Control animals (Control) received equivalent volume of water for injection.

#### Acute toxicity study-according to^42^

Tests were performed on mice of 5 animals per group. In an acute toxicity study conducted on male mice, four different doses of compound **6** were administered intraperitoneally and changes in the appearance of animal skin, mucous membranes, cardiovascular and respiratory disorders and central nervous system activities were observed, as well as mortality during the next 14 days. Particularly, the highest attention was devoted to the appearance of such symptoms as convulsions and tremors, drooling, the appearance of diarrhea, inhibition of the general activity (sedative effect) and collapse of animals into a coma.

#### Anti-inflammatory test

Anti-inflammatory test was performed similarly as in our previous study with a few necessary changes. Briefly, the inflammation was induced in the right hind paw of rats by the topical application of 2 mg/paw of carrageenan dissolved in 0.2 ml of 0.9% saline solution. The rear left paw of the rats, which was used as the control, received the same volume of 0.9% saline solution. Single doses of **6** dissolved in water in the range of 1.0 and 10.0 mg/kg were given intraperitoneally (i.p.) 30 min after carrageenan injection. For comparative purposes (positive control), one group of rats was treated with the single indomethacine (Metindol, inj, 0.06 g/2 ml amp., Pliva Kraków, Poland) in dose of 10 mg/kg, (i.p.) also 30 min after carrageenan administration. The rate of edema of the two paws was measured at 3.0 h after carrageenan injection using a plethysmometer (Hugo Sachs Electronic, Germany).

Change of rat’s paw thickness was evaluated using the following equation: ΔG = (Lc − Lw) − (Rc − Rw) [mL], ΔG—value expressing change in paw’s thickness against baseline (before inflammation), Lw—left paw’s thickness before carrageenan injection, Rw—right paw’s thickness before carrageenan injection, Lc—left paw’s thickness 3.0 h after carrageenan injection, Rc—right paw’s thickness 3.0 h after carrageenan injection^43^.

#### Analgesic activity: tail-flick test

The analgesic effect of the drugs was assessed by the tail-flick test according to^44^ using the apparatus for measuring a nociceptive threshold to infrared heat stimulus on the rat Analgesia—Test (Ugo Basile Tail Flick, Italy). The maximal time of a tale light exposure after drug administration was established as 60 s (cut off). For each rat, before **6** or water administration a preliminary reaction time to the stimulus was established, which was defined as baseline latency. The analgesic effect was assessed after 120 min since the substance treatment. Moreover, for comparative purposes, morphine as a positive control (morphini sulfas 20 mg/ml, Polfarma, Poland) (5.0 mg/kg, s.c.) was administered to rats^44^.

#### Statistical analysis

The data were expressed as means ± SEM and the statistical comparison of results was carried out using ANOVA followed by a Duncan post-hoc test. A p value of < 0.05 was considered as statistically significant.

For Fig. 10. The experimental system demonstrated the existence of variation between the means (ANOVA main effect, F(3,36) = 5.83; p = 0.0023) and indicated the significance of the effect of time (ANOVA effect of time ANOVA II F(1,36) = 75.3; p = 0.0000).

## Supplementary information

Supplementary Information.
