# Supplementary material for: Cytotoxic, analgesic and anti-inflammatory activity of colchicine and its C-10 sulfur containing derivatives
Source: Sci Rep. 2021 Apr 27;11:9034. doi: 10.1038/s41598-021-88260-1 (PMC8079405; doi:10.1038/s41598-021-88260-1)

**Cytotoxic, analgesic and anti-inflammatory activity of colchicine and its C-10 sulfur containing derivatives**

**Joanna Kurek*, Krzysztof Myszkowski, Irena Okulicz-Kozaryn, Agnieszka Kurant, Ewa Kamińska, Michał Szulc, Błażej Rubiś, Mariusz Kaczmarek, Przemysław Ł. Mikołajczak, Marek Murias**

|  |  |
| --- | --- |
| **1. Spectroscopic Study: ^1^H and ^13^C NMR, EI-MS spectra** ……………….……..……  **Fig S1.** Carbon atom numbering in colchicine **1** and **C-10** sulfur derivatives **2-6** | **1**  **1** |
| **1.1. Compound** 10-methylthiocolchicine **2** …..……………………..……….……... | **2** |
| **1.2.** **Compound** 10-Ethylthiocolchicine **3** …………………………….……….…… | **3** |
| **1.3. Compound** 10-*n-*propylthiocolchicine **4** ………………………….…..…….…. | **4** |
| **1.4. Compound** 10-*i*-propylthiocolchicine **5**……………………………………....... | **5** |
| **1.5. Compound** 10-*n*-butylthiocolchicine **6**…………………………………….…… | **6** |
| **2. Impact of tested compounds on Cyclin B1 expression in SKOV-3 cells Fig S2.** ….  **3. Cell cycle analysis** of SKOV-3 cell line cultured in the presence of colchicine, 10-methylthiocolchicine and 10-*n*-butylthiocolchicine at concentrations of 0.1µM, 1.0µM and 10µM after 24 and 72 hours, **FigS3**. | **7**  **8** |

**1. Spectroscopic Study ^1^H and ^13^C NMR**

**Figure S1**. Carbon atom numbering in colchicine **1** and **C-10** sulfur derivatives **2-6**

Obtained 10-alkylthiocolchicine derivatives **2-6** were studied by spectral analysis ^13^C NMR and ^1^H NMR, MS EI, UV, FT-IR and elemental analysis in our previous work [1] were ^1^H and ^13^C NMR spectra of 10-alkilthiocolchicine derivatives were recorded in DMSO-d_6_. ^1^H and ^13^C NMR spectra in present work were recorded in CDCl_3_.

[1] J. Kurek, Wł. Boczoń, M. Murias, K. Myszkowski, T. Borowiak, I. Wolska, Synthesis of sulfur containing colchicine derivatives and their biological evaluation as cytotoxic agents, Let. Drug Des. & Disc. 11, 3, 279–289 (2014).

**1.1. Compound 2 10-methylthiocolchicine**

^13^C NMR (75 MHz, CDCl_3,_ TMS, ppm, 25^o^C) 151.2 (C-1), 125.7 (C-1a), 141.6 (C-2), 153.6 (C-3), 107.3 (C-4), 138.5 (C-4a), 29.9 (C-5), 36.5 (C-6), 52.3 (C-7), 151.7 (C-7a), 128.3 (C-8), 182.4 (C-9), 158.2 (C-10), 126.7 (C-11), 134.8 (C-12), 134.3 (C-12a), 61.6 (C-13), 61.4 (C-14), 56.1 (C-15), 169.9 (C-16), 22.9 (C-17), 15.1 (C-18). ^1^H NMR (300 Hz, CDCl_3,_ TMS, ppm, 25^o^C): 6.5 (HC-4, s), 2.4, 2.5 (HC-5, m), 2.0, 2.3 (HC-6, m), 4.7 (HC-7, m), 7.4 (HC-8, s), 7.3 (HC-11, d), 7.1 (HC-12, d), 3.7 (H_3_C-13, s), 3.9 (H_3_C-14, s), 3.9 (H_3_C-15, s), 7.6 (NH, d), 1.9 (H_3_C-17, s), 2.45 (H_3_C-18, s); Elem. Anal.:Found: C, 63.31; H, 6.00; N, 3.23; S, 7.48 C_22_H_25_NO_5_S requires C, 63.61; H, 6.02; N, 3.37; S, 7.71%], EI MS [M+H^+^] = 415.1.

**1.2. Compound 3 10-Ethylthiocolchicine**

^13^C NMR (75 MHz, CDCl_3,_ TMS, ppm, 25^o^C): 151.15 (C-1), 125.7 (C-1a), 141.6 (C-2), 153.7 (C-3), 107.3 (C-4), 138.6 (C-4a), 29.9 (C-5), 36.4 (C-6), 52.3 (C-7), 151.6 (C-7a), 128.4 (C-8), 182.5 (C-9), 157.3 (C-10), 127.1 (C-11), 134.8 (C-12), 134.4 (C-12a), 61.6 (C-13), 61.4 (C-14), 56.1 (C-15), 169.9 (C-16), 22.8 (C-17), 25.5 (C-18), 12.4 (C-19). ^1^H NMR (300 Hz, CDCl_3,_ TMS, ppm, 25^o^C): 6.5 (HC-4, s), 2.3, 2.5 (HC-5, m), 2.0 2.3 (HC-6, m), 4.7 (HC-7, m), 7.4 (HC-8, s), 7.3 (HC-11, d), 7.2 (HC-12, d), 3.7 (H_3_C-13, s), 3.9 (H_3_C-14, s), 3.9 (H_3_C-15, s), 7.8 (NH, d), 2.0 (H_3_C-17, s), 2.9 (H_2_C-19) 1.5 (H_3_C-18, t); Elem. Anal. for C_23_H_27_NO_5_S, found: C, 64.21; H, 6.25; N, 3.21; S, 7.43 requires C, 64.26; H, 6.29; N,3.25; S, 7.45 %, EI MS [M+H^+^] = 429.1,

**1.3. Compound 4 10-*n*-propylthiocolchicine**

^13^C NMR (75 MHz, CDCl_3,_ TMS, ppm, 25^o^C): 151.2 (C-1), 125.7 (C-1a), 141.7 (C-2), 153.6 (C-3), 107.4 (C-4), 138.5 (C-4a), 29.9 (C-5), 36.6 (C-6), 52.2 (C-7), 151.3 (C-7a), 128.4 (C-8), 182.5 (C-9), 157.6 (C-10), 127.1 (C-11), 134.8 (C-12), 134.3 (C-12a), 61.6 (C-13), 61.4 (C-14), 56.1 (C-15), 169.8 (C-16), 22.9 (C-17), 33.6 (C-20), 21.0 (C-19), 13.8 (C-18). ^1^H NMR (300 Hz, CDCl_3,_ TMS, ppm, 25^o^C): 6.5 (HC-4, s), 2.4, 2.5 (HC-5, m), 2.0 2.2 (HC-6, m), 4.7 (HC-7, m), 7.4 (HC-8, s), 7.3 (HC-11, d), 7.2 (HC-12, d), 3.7 (H_3_C-13, s), 3.9 (H_3_C-14, s), 3.9 (H_3_C-15, s), 7.5 (NH, d), 2.0 (H_3_C-17, s), 2.9 (H_2_C-20, t), 1.8 (H_2_C-19, m) 1.1 (H_3_C-18, t); Elem. Anal. for C_24_H_29_NO_5_S, found: C, 64.93; H, 6.53; N, 3.13; S, 7.20 requires C, 65.00; H, 6.55; N, 3.16; S, 7.22 %, EI MS [M+H^+^] = 443.1,

**1.4. Compound 5 10-*i*-propylthiocolchicine**

^13^C NMR (75 MHz, CDCl_3,_ TMS, ppm, 25^o^C): 151.2 (C-1), 125.7 (C-1a), 141.7 (C-2), 153.6 (C-3), 107.4 (C-4), 138.6 (C-4a), 29.9 (C-5), 36.6 (C-6), 52.2 (C-7), 151.6 (C-7a), 128.7 (C-8), 182.6 (C-9), 156.6 (C-10), 127.8 (C-11), 134.8 (C-12), 134.4 (C-12a), 61.6 (C-13), 61.4 (C-14), 56.1 (C-15), 169.8 (C-16), 22.9 (C-17), 34.6 (C-19), 22.2x2 (C-18). ^1^H NMR (300 Hz, CDCl_3,_ TMS, ppm, 25^o^C): 6.5 (HC-4, s), 2.3, 2.5 (HC-5, m), 2.0 2.3 (HC-6, m), 4.7 (HC-7, m), 7.4 (HC-8, s), 7.3 (HC-11, d), 7.2 (HC-12, d), 3.7 (H_3_C-13, s), 3.9 (H_3_C-14, s), 3.9 (H_3_C-15, s), 7.4 (NH, d), 2.0 (H_3_C-17, s), 2.4 (H_2_C-19, m), 1.5x2 (H_3_C-18, t); Elem. Anal. for C_24_H_29_NO_5_S, found: C,64.93; H, 6.52; N, 3.11; S, 7.18 requires C,65.00; H, 6.55; N, 3.16; S, 7.22 % EI MS [M+H^+^] = 443.3.

**1.5. Compound 6 10-*n*-butylthiocolchicine**

^13^C NMR (75 MHz, CDCl_3,_ TMS, ppm, 25^o^C): 151.1 (C-1), 125.6 (C-1a), 141.5 (C-2), 153.5 (C-3), 107.3 (C-4), 138.6 (C-4a), 29.9 (C-5), 36.2 (C-6), 52.3 (C-7), 151.8 (C-7a), 128.3 (C-8), 182.4 (C-9), 157.5 (C-10), 128.3 (C-11), 134.8 (C-12), 134.4 (C-12a), 61.6 (C-13), 61.3 (C-14), 56.0 (C-15), 170.0 (C-16), 22.7 (C-17), 31.2 (C-21), 29.4 (C-20), 22.3 (C-19), 13.6 (C-18). ^1^H NMR (300 Hz, CDCl_3,_ TMS, ppm, 25^o^C**):** 6.5 (HC-4, s), 2.4, 2.5 (HC-5, m), 2.0, 2.2 (HC-6, m), 4.7 (HC-7, m), 7.5 (HC-8, s), 7.3 (HC-11, d), 7.2 (HC-12, d), 3.7 (H_3_C-13, s), 3.9 (H_3_C-14, s), 3.9 (H_3_C-15, s), 8.2 (NH, d), 1.9 (H_3_C-17, s), 2.9 (H_2_C-21, t), 1.8 (H_2_C-20, m), 1.6 (H_2_C-19, m), 1.0 (H_3_C-18, t); Elem. Anal. for C_25_H_31_NO_5_S, found: C, 65.57; H, 6.30; N, 2.98; S 6.95, requires C, 65.64; H,6.35; N, 3.06; S, 7.00 %, EI MS [M+H^+^] = 457.4, ESI MS [M+H^+^] 458, [M+Na^+^]=480, [M+K^+^]= 496.

**2. Impact of tested compounds on Cyclin B1 expression in SKOV-3 cells**

**(Fig S2)**


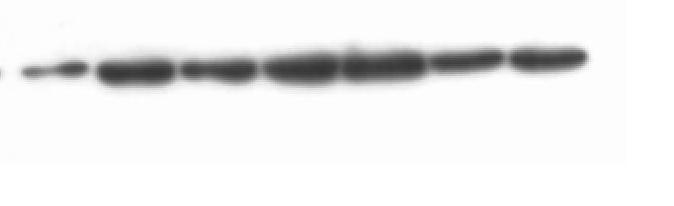

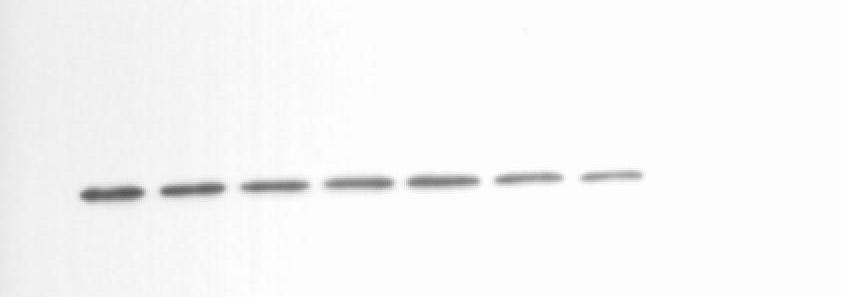

**3. Cell cycle analysis** of SKOV-3 cell line cultured in the presence of colchicine **1**, 10-methylthiocolchicine **2** and 10-*n*-butylthiocolchicine **6**

at concentrations of 0.1µM, 1.0µM and 10µM after 24 and 72 hours, **Fig S3.**


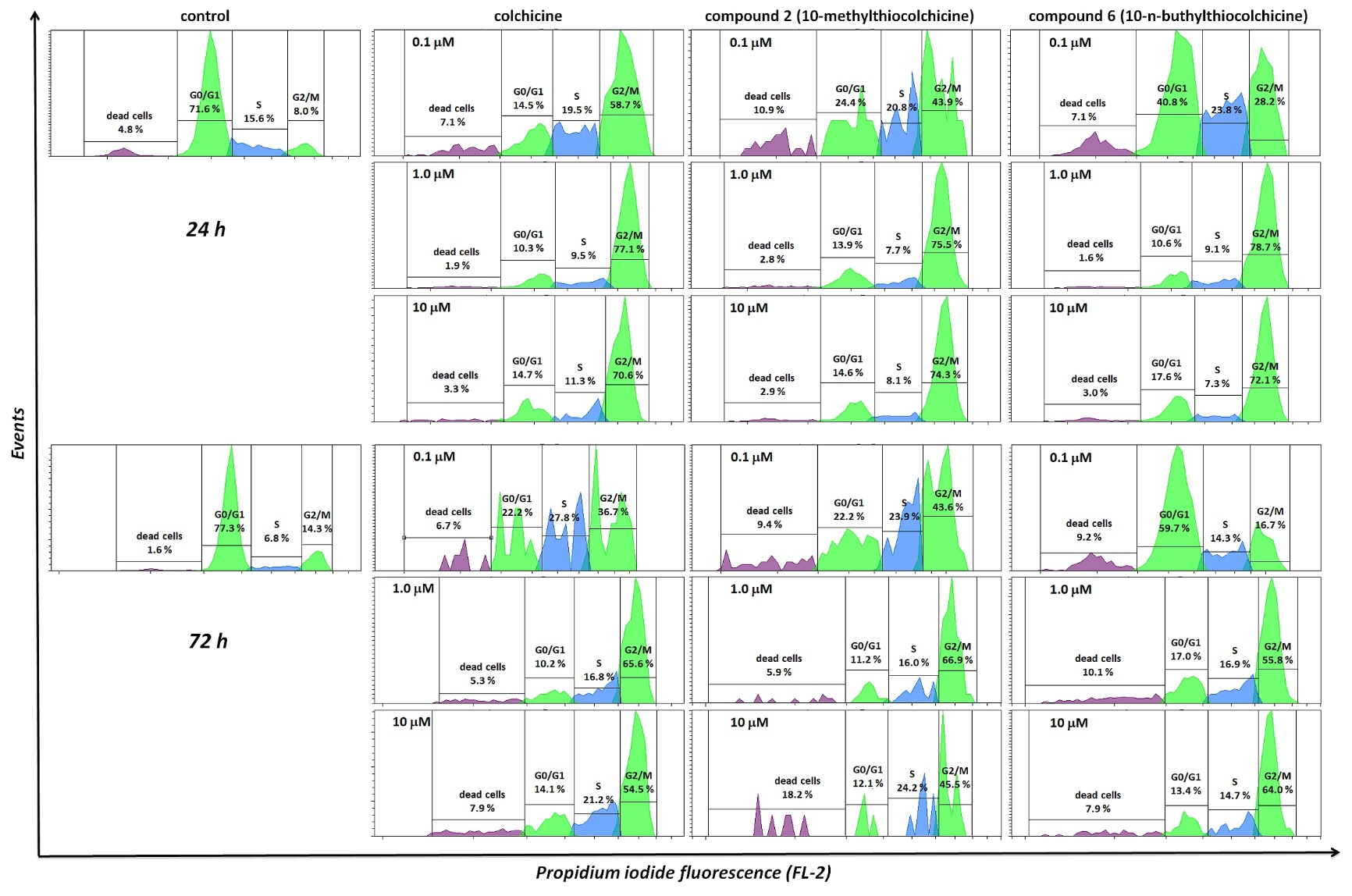

Supplement: Supplementary file 1 — Supplementary Information. [file 41598_2021_88260_MOESM1_ESM.docx]
